# Supplementary material for: Geographic inequities in human papillomavirus vaccine non-uptake and its determinants among adolescent girls in Ethiopia: Evidence from the National Immunization Survey
Source: PLoS One. 2026 Apr 28;21(4):e0348076. doi: 10.1371/journal.pone.0348076 (PMC13123948; doi:10.1371/journal.pone.0348076)
Supplement: S1 File — (DOCX) [file pone.0348076.s002.docx]

AICc----------------------------Akaike Information Criterion corrected

CMHSSH-UOG--------------College of Medicine and Health Sciences Specialized hospital-University of Gondar

DHS-------------------------- Demographic and Health Survey

EA------------------------------Enumeration area

EDHS--------------------------Ethiopian Demographic and Health Survey

ESS-----------------------------Ethiopian Statistical Services

GPS-----------------------------Global positioning system

GWR----------------------------Geographically weighted regression

HPV-----------------------------Human papillomavirus

IDW-----------------------------Inverse distance weighted

MPE-----------------------------Mean predicted error

OLS-----------------------------Ordinary least squares

PCA----------------------------Principal component analysis

RMSE---------------------------Root mean square predicted error

SD-------------------------------Standard deviation

SNNP---------------------------Southern nations, nationalities and peoples of Ethiopia

USA----------------------------Unites states of America

VIF-----------------------------Variance inflation factor

WHO---------------------------World health organization
