# Supplementary material for: Geographic inequities in human papillomavirus vaccine non-uptake and its determinants among adolescent girls in Ethiopia: Evidence from the National Immunization Survey
Source: PLoS One. 2026 Apr 28;21(4):e0348076. doi: 10.1371/journal.pone.0348076 (PMC13123948; doi:10.1371/journal.pone.0348076)
Supplement: Attachment — Submitted filename: PLOS Journal feedback.docx [file pone.0348076.s003.docx]

This paper is very well written. Addresses a very important area to focus on in public health.

I just have minor comments

Human papillomaviruses (HPVs) are a group of epitheliotropic viruses, many of which are

carcinogenic and strongly linked to cervical, anal, oropharyngeal, and other anogenital

cancers.-Kindly include a reference

HPV-related cervical cancer is the world’s most common cancer in women-Add a reference

What are the implications for public health and policy?
