# Supplementary material for: Geographic inequities in human papillomavirus vaccine non-uptake and its determinants among adolescent girls in Ethiopia: Evidence from the National Immunization Survey
Source: PLoS One. 2026 Apr 28;21(4):e0348076. doi: 10.1371/journal.pone.0348076 (PMC13123948; doi:10.1371/journal.pone.0348076)
Supplement: Attachment — Submitted filename: Point-by-point response.docx [file pone.0348076.s004.docx]

## **Point-by-point response**

We sincerely thank the Editor and reviewers for their constructive and insightful comments. Below, we provide a point-by-point response to all comments and suggestions. We have carefully addressed each comment and believe the revisions have strengthened the manuscript. All changes to the manuscript have been highlighted using track changes in the revised manuscript file, with the relevant page and line numbers indicated to locate each revised section.

**Editor**

| Sub-topic | Comments / suggestions raised | Response / justification | Page & line number of the revision |
| --- | --- | --- | --- |
| **Formatting** | | | |
|  | 1. Please ensure that your manuscript meets PLOS ONE's style requirements, including those for file naming. | We sincerely thank the editor for this important reminder. After carefully reviewing the PLOS ONE style templates and formatting guidelines, we have revised the manuscript and supporting documents to align with the journal's formatting guidelines, including:   - Proper formatting of headings and sections - Correct file naming conventions - Appropriate structure as per the journal's templates | Throughout the manuscript and supporting documents |
|  | 2. Thank you for stating the following financial disclosure:   “This work is part of PhD study for KTA and was funded by College of Medicine and Health Sciences, University of Gondar with reference number of PGC//098/12/2016.”  Please state what role the funders took in the study.  If the funders had no role, please state: "The funders had no role in study design, data collection and analysis, decision to publish, or preparation of the manuscript."  If this statement is not correct you must amend it as needed.  Please include this amended Role of Funder statement in your cover letter; we will change the online submission form on your behalf. | Dear editor, thank you for this pointing out this.  We now stated the following in the funding statement section:  The funders had no role in study design, data collection and analysis, decision to publish, or preparation of the manuscript. | Page 15, Line numbers 391–393 |
|  | 3. Thank you for stating the following in the Funding Section of your manuscript:   “This work is part of PhD study for KTA and was funded by College of Medicine and Health Sciences, University of Gondar with reference number of PGC//098/12/2016.”  We note that you have provided funding information that is currently declared in your Funding Statement. However, funding information should not appear in the Acknowledgments section or other areas of your manuscript. We will only publish funding information present in the Funding Statement section of the online submission form.  Please remove any funding-related text from the manuscript and let us know how you would like to update your Funding Statement. Currently, your Funding Statement reads as follows:  Please include your amended statements within your cover letter; we will change the online submission form on your behalf. | We have edited the Acknowledgment section and other areas of the manuscript to avoid funding-related texts. The funding information now appears only in the Funding Statement section of the online submission form. | Page 15, Line numbers 376–382 |
|  | 4. When completing the data availability statement of the submission form, you indicated that you will make your data available on acceptance. We strongly recommend all authors decide on a data sharing plan before acceptance, as the process can be lengthy and hold up publication timelines. Please note that, though access restrictions are acceptable now, your entire data will need to be made freely accessible if your manuscript is accepted for publication. This policy applies to all data except where public deposition would breach compliance with the protocol approved by your research ethics board. If you are unable to adhere to our open data policy, please kindly revise your statement to explain your reasoning and we will seek the editor's input on an exemption. Please be assured that, once you have provided your new statement, the assessment of your exemption will not hold up the peer review process. | Thank you. We confirm that we will make all data underlying the findings fully available. We have updated our Data Availability Statement as follows:  All relevant data are within the manuscript and its Supporting Information file. | Page 15; line number 388 |
|  | 5. We note that Figures 3, 4, 5, 6, 7, and 8 in your submission contain [map/satellite] images which may be copyrighted.   We require you to either (1) present written permission from the copyright holder to publish these figures specifically under the CC BY 4.0 license, or (2) remove the figures from your submission:  Please upload the completed Content Permission Form or other proof of granted permissions as an "Other" file with your submission.  In the figure caption of the copyrighted figure, please include the following text: “Reprinted from [ref] under a CC BY license, with permission from [name of publisher], original copyright [original copyright year].”  b. If you are unable to obtain permission from the original copyright holder to publish these figures under the CC BY 4.0 license or if the copyright holder’s requirements are incompatible with the CC BY 4.0 license, please either i) remove the figure or ii) supply a replacement figure that complies with the CC BY 4.0 license. Please check copyright information on all replacement figures and update the figure caption with source information. If applicable, please specify in the figure caption text when a figure is similar but not identical to the original image and is therefore for illustrative purposes only. | Thank you for bringing this important copyright issue to our attention.   The administrative boundary shapefiles used in this study were obtained from the Humanitarian Data Exchange (HDX) platform (<https://data.humdata.org/dataset/cod-ab-eth>), which provides open-access data publicly available for unrestricted use. | Figures 3–8 and corresponding captions |
|  | 6. Please include a separate caption for each figure in your manuscript. | Thank you for this reminder. We have ensured that each figure in the manuscript now has its own descriptive caption placed immediately below the figure. The captions provide sufficient detail for readers to understand the figure content without referring to the main text. | Figures 1–8 and corresponding captions |
|  | 7. If the reviewer comments include a recommendation to cite specific previously published works, please review and evaluate these publications to determine whether they are relevant and should be cited. There is no requirement to cite these works unless the editor has indicated otherwise. | Not applicable because neither of the reviewers recommended citing specific previously published works. |  |
|  | 8. Please review your reference list to ensure that it is complete and correct. If you have cited papers that have been retracted, please include the rationale for doing so in the manuscript text, or remove these references and replace them with relevant current references. Any changes to the reference list should be mentioned in the rebuttal letter that accompanies your revised manuscript. If you need to cite a retracted article, indicate the article’s retracted status in the References list and also include a citation and full reference for the retraction notice. | We have thoroughly reviewed our reference list and confirm that:   - All references are complete and correctly formatted according to PLOS ONE style - No retracted articles are cited | Page 16–20; line number 399–556 |

## **Reviewer 1:**

| Sub-topic | Comments / suggestions raised | Response / justification | Page & line number of the revision |
| --- | --- | --- | --- |
| **Introduction** | | | |
| **Overall** | This paper is very well written. Addresses a very important area to focus on in public health. I just have minor comments | - Thank you for your positive assessment of our work. We appreciate your recognition of the importance of this research area. We have carefully addressed each of your minor comments below. |  |
| **Introduction, 1^st^ paragraph** | Human papillomaviruses (HPVs) are a group of epitheliotropic viruses, many of which are carcinogenic and strongly linked to cervical, anal, oropharyngeal, and other anogenital cancers.-Kindly include a reference | Thank you for this suggestion. We have added appropriate references to support this statement. | Page 3, Line number 63 |
| **Introduction, 1^st^ paragraph** | HPV-related cervical cancer is the world’s most common cancer in women-Add a reference | Thank you for your observation. We have put specific reference for the indicated statement. | Page 3, Line number 64 |
|  |  |  |  |
| **Discussion** | | | |
|  | What are the implications of the findings for public health and policy? | Thank you for raising this important point. We recognize that while we attempted to discuss implications in the concluding sentence of each paragraph throughout the Discussion section, this aspect could be strengthened. In response, we have revised the Discussion to more explicitly address the public health and policy implications of our findings. | Page 12–14, Line numbers 286–288, 296–300, 312–315, 326–329, 341–343,  353–356 |

**Reviewer 2**

| Sub-topic | Comments / suggestions raised | Response / justification | Page & line number of the revision |
| --- | --- | --- | --- |
|  | | | |
| **Overall** | The article contains most of the requirements necessary for good publication. It has scientific relevance and was based on strong references. It presents results and conclusions in accordance with the proposed objectives. | Thank you for your positive assessment of our work. We appreciate your recognition of its scientific relevance and robust methodological approach. We have carefully addressed your specific suggestions below. |  |
| **Method** | | | |
|  | "I suggest including in the methods section which official reference was used to establish the age of adolescence. In the present study, it cites 14 years or older and it was left vague. It is known that there are different minimum and maximum age standards. Depending on the location, there are differences between the minimum and maximum age for teenagers. This can modify some data." | Thank you for raising this important point. We acknowledge that the initial description of "14 years or older" was unclear; we have now revised it to 15–18 years. This cut-off was determined by the age distribution in the dataset. Although the World Health Organization defines adolescents as individuals aged 10–19 years, our study focuses specifically on girls aged 15–18 years. This was due to the nature of the secondary data, which were originally collected from adolescent girls eligible for HPV vaccination under a single-age cohort strategy targeting only 14-year-olds. As data were collected retrospectively, one year after the vaccination campaign, participants who were 14 at the time of vaccination had turned 15 by the time of data collection. Therefore, 15 years was used as the lower age limit, while the upper age limit (18 years) was determined by the maximum age observed in the dataset. | Page 4, Line numbers 108–116 |
|  | Another point to detail is what standard was used to differentiate between levels of wealth and poverty, since this variable was significant in the study. | Thank you for raising this concern. We agree that clarity regarding the construction of the wealth index is essential for interpreting the findings. We have provided detailed information about the calculation of the wealth index as follows:  The household wealth index was constructed using principal component analysis (PCA) [27]. The index incorporated housing characteristics (flooring, roofing, wall materials, water source, toilet facility, and cooking fuel) and asset ownership (television, radio, refrigerator, bicycle, motorcycle, car, mobile telephone, agricultural land, and livestock). PCA generated factor scores, with the first principal component used to create the wealth index, ranking households into quintiles from poorest to wealthiest. For our analysis, we categorized households into five wealth levels (poorest, poorer, middle, richer, and richest), following standard DHS practice to enable cross-study comparisons. | Page 6, Line numbers 158–166 |

We once again thank the editor and reviewers for their insightful and constructive feedback, which has significantly improved the clarity, rigor, and overall quality of the manuscript.
